# Version 3 of the Global Aridity Index and Potential Evapotranspiration Database

**DOI:** 10.1038/s41597-022-01493-1

**Published:** 2022-07-15

**Authors:** Robert J. Zomer, Jianchu Xu, Antonio Trabucco

**Affiliations:** 1 Centre for Mountain Futures, Kunming Institute of Botany, Chinese Academy of Science, Kunming, 650201 Yunnan China; 2 CIFOR-ICRAF China Program, World Agroforestry (ICRAF), Kunming, China; 3 Euro-Mediterranean Center on Climate Change, IAFES Division, Sassari, Italy

**Keywords:** Hydrology, Hydrology, Agroecology, Ecosystem ecology, Ecological modelling

## Abstract

The “Global Aridity Index and Potential Evapotranspiration Database - Version 3” (Global-AI_PET_v3) provides high-resolution (30 arc-seconds) global hydro-climatic data averaged (1970–2000) monthly and yearly, based upon the FAO Penman-Monteith Reference Evapotranspiration (ET_0_) equation. An overview of the methods used to implement the Penman-Monteith equation geospatially and a technical evaluation of the results is provided. Results were compared for technical validation with weather station data from the FAO “CLIMWAT 2.0 for CROPWAT” (*ET*_0_*: r*^2^ = 0*.85; AI: r*^2^ = *0.90*) and the U.K. “Climate Research Unit: Time Series v 4.04” (*ET*_*0*_*: r*^2^ = 0*.89;* AI: *r*^2^ = 0*.83*), while showing significant differences to an earlier version of the database. The current version of the Global-AI_PET_v3 supersedes previous versions, showing a higher correlation to real world weather station data. Developed using the generally agreed upon standard methodology for estimation of reference ET_0_, this database and notably, the accompanying source code, provide a robust tool for a variety of scientific applications in an era of rapidly changing climatic conditions.

## Abstract (summary)

**Table:** 

Measurement(s)	evapotranspiration
Technology Type(s)	Geographic Information System
Sample Characteristic - Environment	climate system

## Background & Summary

Potential evapotranspiration (PET) is a measure of the ability of the atmosphere to remove water through evapotranspiration (ET)^1,2^, and is the sum of two processes, evaporation and transpiration, which transfer water from the land surface to the atmosphere. These two processes occur simultaneously, with the rates of both dependent on solar radiation, air temperature, relative humidity (i.e., vapor pressure deficit) and wind speed^3,4^, as well as specific crop characteristics and cultivation practices^2^. Measures of, and indices based upon PET (or the ET of a reference crop under optimal conditions) are widely used in a range of scientific disciplines and practical applications, particularly in agricultural and natural resource management, where it is applied at scales from farm to regional and global^5,6^. In a rapidly changing global environment and climate, these metrics, and their derivative indices, become a direct and critical measure, and predictive tool, of the trend, direction, and magnitude of climatic change, and it’s impacts upon the terrestrial biosphere, with implications for plant growth, sustainable development, and eventually, considering the recently released conclusions of the latest IPCC^7,8^ reports, for human civilization.

Likewise, aridity is a complex concept that ideally requires a comprehensive assessment of hydro-climatological and hydro-ecological variables to fully describe or understand anticipated changes. A widely used approach to assess status and changes in aridity is the aridity index (AI), defined as the ratio of precipitation to PET. Aridity indices^9–11^ provide a measure of moisture availability for potential growth of reference crop or other specific vegetation types^1,12,13^. Summarizing the aridity concept into a single number, the use of aridity indices allows for both spatial and temporal comparisons and provide an important baseline for measuring and anticipating the impacts of climatic change. The AI reflects the exchanges of energy and water between the land surface and the atmosphere, and its variation can be used as input for a variety of operational decision making, such as irrigation and crop management, as well as forecasting drought and flood patterns, which makes it of great significance for agricultural production and water management^14^.

The first version of the “Global Aridity Index and PET Database” (Global-AI_PET_v1)^15^, using the global climatic dataset WorldClim (version 1.4: 1960–1990) has been available online since 2009^15–18^, and a subsequent version the “Global Aridity Index and Potential Evapotranspiration (ET_0_) Climate Database” (Global-AI_PET_v2)^19^ implementing a Penman-Monteith equation and based on the updated WorldClim version 2.0^20^ (1970–2000), has been available online since 2019. These datasets been downloaded currently in excess of 47,000 times, and applied across a wide range of disciplines, with nearly 1500 citations on topics ranging from agricultural and natural resource science, to genetics, anthropology, archaeology, conflict resolution, and climate change. It has been found useful in a wide variety of applications, particularly related, but not limited to water management^21,22^ and crop production, but also socio-ecological and socio-economic applications related to sustainable development^23,24^, climate change impacts^25,26^, and adaptation^27,28^. The topics of papers citing this dataset range from global environmental stratification^29–31^, to human migration^32^, pastoralism and dryland environmental threats^33,34^, wildlife and restoration ecology^35^, fire modeling^36^, child mortality^37^, and epidemiological^38–40^ and other human and livestock health research^41–45^, such as the effect of malaria control^39,40^, or mapping the zoonotic niche of Ebola virus disease in Africa^38^.

This paper describes the updated Version 3 of the “Global Aridity Index and Potential Evapotranspiration (ET_0_) Database” (Global-AI_PET_v3)^46^, which is based upon a fully parameterized geospatial implementation of the FAO-56 Penman-Monteith equation (referred to hereafter as “FAO-56”). An overview of the methods used to implement FAO-56 geospatially on a per grid cell basis, and a technical evaluation of the results, both in relation to weather station data, and in comparison, with the two previous versions (Global-AI_PET_v1/Global-AI_PET_v2) is provided as guidance to previous users. Results are compared for technical validation with weather station data from the FAO “CLIMWAT 2.0 for CROPWAT”^47^ and the global gridded time series data from the CRU_TS (version 4.04)^48^.

The updated Global-AI_PET_v3^46^ database is archived and available online for download at: 10.6084/m9.figshare.7504448.v5.

## Methods

### Calculating Potential Evapotranspiration using Penman-Monteith

Among several equations used to estimate PET, an implementation of the Penman-Monteith equation originally presented by the Food and Agriculture Organization FAO-56^1^, is considered a standard method^3,12,13,49^. FAO-56^1^ defined PET as the ET of a reference crop (ET_0_) under optimal conditions, in this case with the specific characteristics of well-watered grass with an assumed height of 12 centimeters, a fixed surface resistance of 70 seconds per meter and an albedo of 0.23^1^. Less specifically, “reference evapotranspiration”, generally referred to as “ET_0_”, measures the rate at which readily available soil water is evaporated from specified vegetated surfaces^2,13^, i.e., from a uniform surface of dense, actively growing vegetation having specified height and surface resistance, not short of soil water, and representing an expanse of at least 100 m of the same or similar vegetations^1,13^. ET_0_ is one of the essential hydrological variables used in many research efforts, such as study of the hydrologic water balance, crop yield simulation, irrigation system management and in water resources management, allowing researchers and practitioners to study the evaporative demand of the atmosphere independent of crop type, crop development and management practices^2,4,13,49^. ET_0_ values measured or calculated at different locations or in different seasons are comparable as they refer to the ET from the same reference surface. The factors affecting ET_0_ are climatic parameters, and crop specific resistances coefficients solved for reference vegetation. Other crop specific coefficients (K_c_) may then be used to determine the ET of specific crops (ET_c_), and which can in turn be determined from ET_0_^1^.

As the Penman-Monteith methodology is predominately a climatic approach, it can be applied globally as it does not require estimations of additional site-specific parameters. However, a major drawback of the Penman-Monteith method is its relatively high need for specific data for a variety of parameters (i.e., windspeed, relative humidity, solar radiation). Zomer *et al*.^18^ compared five methods of calculating PET with parameters from data available at the time and settled upon using a Modified Hargreaves-Thornton equation^50^ which required less parametrization to produce the Global-AI_PET_v1^16–18^. Several other attempts to produce global PET datasets with concurrently available global datasets came to similar conclusions^51–53^. The Modified Hargreaves-Thornton method required less parameterization with relatively good results, relying on datasets which were available at the time for a globally applicable modeling effort. The Global-AI_PET_v1 used the WorldClim_v1.4^20^ downscaled climate dataset (30 arcseconds; averaged over the period 1960–1990) for input into the global geospatial implementation of the Modified Hargreaves-Thornton equation, applied on a per grid cell basis at approximately 1 km resolution (30 arcseconds). More recently, the UK Climate Research Unit released the “CRU_TS Version 4.04”, which now includes a Penman-Monteith calculated PET (ET_0_) global coverage, however at a relatively coarse resolution of 0.5 × 0.5 degrees. A number of satellite-based remote sensing datasets^22,54–57^ are now available and in use to provide the parameters for ET_0_ estimates, in some cases providing high spatial and/or temporal resolution and are likely to become increasingly utilized as the historical data record lengthens and sensors improve.

The latest 2.0 versions of WorldClim^58^ (currently version 2.1; released January 2020), in addition to being updated with improved data and analysis, and a revised baseline (1970–2000), includes several additional primary climatic variables, beyond temperature and precipitation, namely: solar radiation, wind speed and water vapor pressure. The addition of these variables allowed that the global data now available was sufficient to effectively parameterize the FAO-56 equation to estimate ET_0_ globally at the 30 arc seconds scale (~1 km at equator).

The FAO-56 Penman-Monteith equation, described in detail below, has been implemented on a per grid cell basis at 30 arc seconds resolution, using the Python programming language (version 3.2). The data to parametrize the various components equations required to arrive at the ET_0_ estimate were obtained from the Worlclim 2.1^58^ climatological dataset, which provides values averaged over the time period 1970–2000 for minimum, maximum and average temperature; solar radiation; wind speed, and water vapor pressure. Subroutines in the program include calculation of the psychrometric constant (aerodynamic resistance), saturation vapor pressure, vapor pressure deficit, slope of vapour pressure curve, air density at constant pressure, net shortwave radiation at crop surface, clear-sky solar radiation, net longwave radiation at crop surface, net radiation at the crop surface, and the calculation of daily and monthly ET_0_. This process is described below. Geospatial processing and analysis were done using ArcGIS Pro v 2.9 (ESRI, 2020), Python (ArcPy) programming language (version 3.2), and Microsoft Excel for further data analysis, graphics and presentation.

### Global Reference Evapotranspiration (Global-ET_0_)

Penman^59^, in 1948, first combined the radiative energy balance with the aerodynamic mass transfer method and derived an equation to compute evaporation from an open water surface from standard climatological records of sunshine, temperature, humidity and wind speed. This combined approach eliminated the need for the parameter “most difficult” to measure, surface temperature, and allowed for the first time an opportunity to make theoretical estimates of ET from standard meteorological data. Consequently, these estimates could also now be made retrospectively. This so-called combination method was further developed by many researchers and extended to cropped surfaces by introducing resistance factors. Among the various derivations of the Penman equation is the inclusion of a bulk surface resistance term^60^, with the resulting equation now called the Penman-Monteith equation^3^, as standardized in FAO-56^1^ and subsequently by the American Society of Civil Engineers - Technical Committee on Standardization of Reference Evapotranspiration^12,13,49,61^. The FAO-56 Penman-Monteith form of the combination equation to estimate ET_0_ is calculated as:

1 $$ETo=\frac{\Delta \left({R}_{n}-G\right)+{\rho }_{a}{c}_{p}\frac{({e}_{s}-{e}_{a})}{{r}_{a}}}{\Delta +\gamma \left(1+\frac{{r}_{s}}{{r}_{a}}\right)}$$

Where

*ET*_0_ is the evapotranspiration for reference crop, as mm day^−1^

*R*_*n*_ is the net radiation at the crop surface, as MJ m^−2^ day^−1^

*G* is the soil heat flux density, as MJ m^−2^ day^−1^

*c*_*p*_ is the specific heat of dry air

*p*_*a*_ is the air density at constant pressure

*e*_*s*_ is the saturation vapour pressure, as *kPa*

*e*_*a*_ is the actual vapour pressure, as *kPa*

*e*_*s*_ - e_a_ is the saturation vapour pressure deficit, as *kPa*

$\Delta$ is the slope vapour pressure curve, as *kPa* °C^−1^

$\gamma$ is the psychrometric constant, as *kPa* °C^−1^

*r*_*s*_ is the bulk surface resistance, as m s^−1^

*r*_*a*_ is the aerodynamic resistance, as m s^−1^

### Psychrometric Constant (γ)

The Atmospheric Pressure (*Pr, [KPa])* is the pressure exerted by the weight of the atmosphere and is thus dependent on elevation (elev, [m]). To a certain (and limited) extent evaporation is promoted at higher elevations:

2 $$Pr=101.3\ast {\left(\frac{293-0.0065\ast elev}{293}\right)}^{5.26}$$

Instead, the psychrometric constant, [*γ*, *kPa C*^*−1*^] is expressed as:

3 $$\gamma =\frac{{c}_{p}\ast Pr}{\varepsilon \ast \lambda }=\frac{0.001013\ast Pr}{0.622\ast 2.45}$$

Where *c*_*p*_ is the specific heat at constant pressure [MJ kg^−1^ °C^−1^] and is equal to 1.013 10^−3^, λ is the latent heat of vaporization [MJ kg^−1^] and is equal to 2.45, while ε is the molecular weight ratio between water vapour and dry air and is equal to 0.622.

Elevation data has been obtained from the Shuttle Radar Topography Mission (SRTM) aggregated to 30 arc-second spatial resolution^62^ and combined with the USGS GTOPO30^63^ database for the areas north of 60°N and south of 60°S where no SRTM data was available (available at https://worldclim.org).

### Air Density at Constant Pressure [ρ_*a*_]

The mean Air Density at Constant Pressure [ρ_a_, Kg m^−3^] can be represented as:

4 $${\rho }_{a}=\frac{Pr}{{T}_{Kv}\ast R}$$

While *R* is the specific heat constant (0.287, KJ Kg^−1^ K^−1^), the virtual temperature *T*_*Kv*_ can be represented as well as:

5 $${T}_{Kv}=1.01\ast ({T}_{avg}+273)$$

With T_avg_ as the mean daily air temperature at 2 m height [C°].

### Saturation Vapor Pressure [*KPa*]

Saturation Vapor Pressure [KPa] is strictly related to temperature values (*T*)

6 $${e}_{s\_T}=0.6108\ast ex{p}^{\left[\frac{17.27\ast T}{T+237.3}\right]}$$

Values of saturation vapor pressures, as function of temperature, are calculated for both Minimum Temperature [*T*_*min*_, C°] and Maximum temperature [*T*_*max*_, C°]. Due to nonlinearity of the equation, the mean saturation vapour pressure [*e*_*s*_*, KPa*] is calculated as the average of saturation vapour pressure at minimum [*e*_*s_min*_] and maximum temperature [*e*_*s_max*_]

7 $${e}_{s}=\frac{{e}_{s\_Tmax}+{e}_{s\_Tmin}}{2}$$

The actual vapour pressure [*e*_*a*_*, KPa*] is the vapour pressure exerted by the water in the air and is usually calculated as function of Relative Humidity [*RH*]. Water vapour pressure is already available as one of the Worldclim 2.1 variables.

8 $${e}_{a}=RH/100\,\ast \,{e}_{s}$$

The vapour pressure deficit (*e*_*s*_-*e*_*a*_), [*KPa*] is the difference between the saturation (*es*) and actual vapour pressure (${e}_{a}$).

### Slope of Saturation Vapor Pressure (Δ)

The Slope of Saturation Vapor Pressure [Δ, *kPa C*^*−1*^] at a given temperature is given as function of average temperature:

9 $$\Delta =\frac{4098\ast 0.6108\,ex{p}^{\left(\frac{17.27\ast {T}_{avg}}{{T}_{avg}+237.3}\right)}}{{\left({T}_{avg}+237.3\right)}^{2}}$$

Where *T*_*avg*_ [C°] is the average temperature.

### Net Radiation At The Crop Surface (*R*_*n*_)

Net radiation [*R*_*n*_, MJ m^−2^ day^−1^] is the difference between the net shortwave radiation [*Rns*, MJ m^−2^ day^−1^] and the net longwave radiation [*R*_*nl*_, MJ m^−2^ day^−1^], and is calculated using solar radiation (*R*_*s*_). In Worldclim 2.1 solar radiation (*R*_*s*_) is given as KJ m^−2^ day^−1^. Thus, for computation of ET_0_, its unit should be converted to MJ m^−2^ day^−1^ and thus its value should be divided by 1000. The net accounting of either longwave and shortwave radiation sums up the incoming and outgoing components.

10 $${R}_{n}={R}_{ns}-{R}_{nl}$$

The net shortwave radiation [*R*_*ns*_, MJ m^−2^ day^−1^] is the fraction of the solar radiation *R*_*s*_ that is not reflected from the surface. The fraction of the solar radiation reflected by the surface is known as the albedo [α]. For the green grass reference crop, α is assumed to have a value of 0.23. The value of *Rns* is:

11 $${R}_{ns}={R}_{s}\,\ast \,(1-\alpha )$$

The difference between outgoing and incoming longwave radiation is called the net longwave radiation [*R*_*nl*_]. As the outgoing longwave radiation is almost always greater than the incoming longwave radiation, *R*_*nl*_ represents an energy loss. Longwave energy emission is related to surface temperature following Stefan-Boltzmann law. Thus, longwave radiation emission is calculated as positive in the outward direction, while shortwave radiation is positive in the downward direction. The net energy flux leaving the earth’s surface is influenced as well by humidity and cloudiness

12 $${R}_{nl}=\sigma \ast \left(\frac{{T}_{max,\,K}^{4}+{T}_{min,\,K}^{4}}{2}\right)\ast \left(0.34-0.14\ast \sqrt{{e}_{a}}\right)\ast \left(1.35\ast \frac{{R}_{s}}{{R}_{so}}-0.35\right)$$

Where σ represent the Stefan-Boltzmann constant (4.903 10-9 MJ K^−4^ m^−2^ day^−1^), *T*_*max*,K_ and *T*_*min*,K_ the maximum and minimum absolute temperature (in Kelvin; K = C° + 273.16), e_a_ is the actual vapour pressure; *R*_*s*_ the measured solar radiation [MJ m^−2^ day^−1^] and *R*_*so*_ is the calculated clear-sky radiation [MJ m^−2^ day^−1^]. *R*_*so*_ is calculated as function of extraterrestrial solar radiation [*R*_*a*_, MJ m^−2^ day^−1^**]** and elevation (*elev*, m):

13 $${R}_{so}={R}_{a}\ast (0.75+0.00002\ast elev)$$

The extraterrestrial radiation, [*R*_*a*_, MJ m^−2^ day^−1^], is estimated from the solar constant, solar declination and day of the year. It requires specific information about latitude and Julian day to accomplish a trigonometric computation of the amount of solar radiation reaching the top of the atmosphere following trigonometric computations as shown in Allen *et al*.^1^.

Although the soil heat flux is small compared to *R*_*n*_, particularly when the surface is covered by vegetation, changes of soil heat flux may still be relevant at monthly scale. However, accurate assessments of soil heat flux may require computation of soil heat capacity, related to its mineral composition and water content, which in turn may be rather inaccurate at global scale at resolution of 30 arc sec. Thus, for simplicity, changes in soil heat fluxes are ignored (*G* = 0).

### Bulk Surface Resistance (*r*_*s*_)

The resistance nomenclature distinguishes between aerodynamic resistance and surface resistance factors. The surface resistance parameters are often combined into one parameter, the ‘bulk’ surface resistance parameter which operates in series with the aerodynamic resistance. The surface resistance, *r*_*s*_, describes the resistance of vapour flow through stomata openings, total leaf area and soil surface. The aerodynamic resistance, ra, describes the resistance from the vegetation upward and involves friction from air flowing over vegetative surfaces. Although the exchange process in a vegetation layer is too complex to be fully described by the two resistance factors, good correlations can be obtained between measured and calculated evapotranspiration rates, especially for a uniform grass reference surface.

A general equation for the bulk surface resistance (*r*_*s*_, [s m^−1^]) describes a ratio between the bulk stomatal resistance of a well illuminated leaf (r_l_) and the active sunlit leaf area of the vegetation:

14 $${r}_{s}=\frac{{r}_{l}}{LA{I}_{active}}$$

The stomatal resistance of a single leaf under well-watered conditions has a value of about 100 s m^−1^. It can be assumed that about half (0.5) of the total LAI is actively contributing to vapour transfer, while it can also be roughly generalized that for short crops there is a linear relation between LAI and crop height (*h*):

15 $$LAI=24\ast h$$

When the evapotranspiration simulated with the Penman-Monteith method is referred to a specific reference crop, denoted as ET_0_, a simplified computation of the method can occur that defines a priori specific variables into constant values. In this case, the reference surface is a hypothetical grass reference crop, well-watered grass of uniform height, actively growing and completely shading the ground, with an assumed crop height of 0.12 m, and an albedo of 0.23. The surface resistance for this hypothetical grass can be simplified to the following:

16 $${r}_{s}=\frac{100}{0.5\ast 24\ast h}$$

For such reference crop the surface resistance is fixed to 70 s m^−1^ and implies a moderately dry soil surface resulting from about a weekly irrigation frequency.

### Aerodynamic Resistance (*r*_*a*_)

The aerodynamic resistance [s m^−1^] verifies the transfer of water vapour and heat from the vegetation surface into the air, and is controlled by both vegetation status but also atmospheric turbulence under theoretical aspect as:

17 $${r}_{a}=\frac{ln\left[\frac{{z}_{m}-d}{{z}_{om}}\right]\ast ln\left[\frac{{z}_{h}-d}{{z}_{oh}}\right]}{{k}^{2}{u}_{z}}$$

Z_m_ [m] is the height [*h*] of wind measurements and Z_h_ [m] is the height of humidity measurements. These are normally set at 2 meters height, although several climate models may provide them for higher heights (e.g. 10 m). The zero plane displacement (d [m]) term can be estimated as two thirds of crop height, while Z_om_ is the roughness length governing momentum transfer, and can be calculated as Z_om_ = 0.123 * *h*.

The roughness length governing transfer of heat and vapour, Z_oh_ [m], can be approximated as one tenth of Z_om_. *k* is the von Karman’s constant, equal to 0.41, and *u*_*z*_ [m s-1] is the wind speed at height *z*.

The reference surface, as stated, is a hypothetical grass reference crop, well-watered grass of uniform height, actively growing and completely shading the ground, with an assumed crop height of 0.12 m, and an albedo of 0.23. For such reference crop the surface resistance is fixed to 70 s m-1 and implies a moderately dry soil surface resulting from about a weekly irrigation frequency.

When crop height is equal to 0.12 and wind/humidity measurements are taken at 2 meters height, then the aerodynamic resistance can be simplified as:

18 $${r}_{a}=\frac{208}{{u}_{2}}$$

### Reference Evapotranspiration (*ET*_*0*_)

Given the above, and the specific properties of the standard reference crop, the FAO-56 Penman-Monteith method to estimate ET_0_ then can be calculated as:

19 $$ETo=\frac{0.408\ast \Delta \ast \left({R}_{n}-G\right)+\gamma \frac{900}{{T}_{avg}+273}\ast {u}_{2}\ast \left({e}_{s}-{e}_{a}\right)}{\Delta +\gamma \left(1+\frac{{r}_{s}}{{r}_{a}}\right)}$$

### Aridity Index (*AI*)

Aridity is often expressed as a generalized function of precipitation and PET. The ratio of precipitation over PET (or ET_0_). That is, the precipitation available in relation to atmospheric water demand^64^ quantifies water availability for plant growth after ET demand has been met, comparing incoming moisture totals with potential outgoing moisture^65^.

Geospatial analysis and global mapping of the AI for the averaged 1970–2000 time period has been calculated on a per grid cell basis, as:

20 $$Al=MA\_Prec/MA\_E{T}_{0}$$

where:

*AI* = Aridity Index

*MA_Prec* = Mean Annual Precipitation

*MA_ET*_*0*_ = Mean Annual Reference Evapotranspiration

Mean annual precipitation (*MA_Prec*) values were obtained from the WorldClim v 2.1^58^, as averaged over the period 1970–2000, while ET_0_ datasets estimated on a monthly average basis by the Global-ET_0_ (i.e., modeled using the method described above) were aggregated to mean annual values (*MA_ET*_*0*_). Using this formulation, AI values are unitless, increasing with more humid condition and decreasing with more arid conditions.

As a general reference, a climate classification scheme for Aridity Index values provided by UNEP^64^ provides an insight into the climatic significance of the range of moisture availability conditions described by the AI.

**Table:** 

**Aridity Index Value**	**Climate Class**
<0.03	Hyper Arid
0.03–0.2	Arid
0.2–0.5	Semi-Arid
0.5–0.65	Dry sub-humid
>0.65	Humid

## Data Records

The Reference Evapo-Transpiration (Global-ET_0_) and Aridity Index (Global-AI) datasets included in the Global-AI_PET_v3 Database provide high-resolution (30 arc-seconds) global raster climate data for the 1970–2000 period, related to evapo-transpiration processes and rainfall deficit for potential vegetative growth, based upon implementation of a Penman-Monteith Reference Evapo-transpiration (ET_0_) equation. Dataset files include the following geospatial raster datasets (distributed online in GEOTIFF format) covering the entire world:

### Global-ET_**0**_

Geospatial raster datasets are available as monthly averages (12 data layers, i.e., one layer for each month) or as an annual average (1 dataset) for the 1970–2000 period, plus the standard deviation of the annual average (1 dataset).

### Global-AI

Geospatial raster datasets are available as monthly averages (12 data layers, i.e. one layer for each month) or as an annual average (1 data layer) for the 1970–2000 period.

**Table:** 

Prefix is either:	
ai_v3_	Global-AI datasets
et0_v3_	Global- ET0 datasets
Suffix is either:	
01, 02, … 12	month of the year
yr	mean annual
yr_	sdstandard deviation of the mean annual
Examples:	
ai_v3_yr	is the mean annual AI
et0_v3_02	is the mean monthly ET0 for the month of February
et0_v3_yr	is the mean annual ET0
eto_v3_yr_sd	is the standard deviation of the mean annual ET0

The ET_0_ geodataset values are defined as the total mm of ET_0_ per month or per year.

The AI values reported in the GeoTIFF (.tif) files have been multiplied by a factor of 10,000 to derive and distribute the data as integers (with 4 decimal accuracy). This multiplier has been used to increase the precision of the variable values without using decimals (real or floating values are less efficient in terms of computing time and space compared to integer values). The AI values in the GeoTIFF (.tif) files need to be multiplied by 0.0001 to retrieve the values in the correct units.

The geospatial dataset is in geographic coordinates; datum and spheroid are WGS84; spatial units are decimal degrees. The spatial resolution is 30 arc-seconds or 0.008333 degrees.

The ET_0_ and AI dataset have been processed and finalized in GeoTIFF data format. These rasters have been zipped (.zip) into monthly series or individual annual layers available for online access at: 10.6084/m9.figshare.7504448.v5^46^.

## Technical Validation

**Fig. 1:**
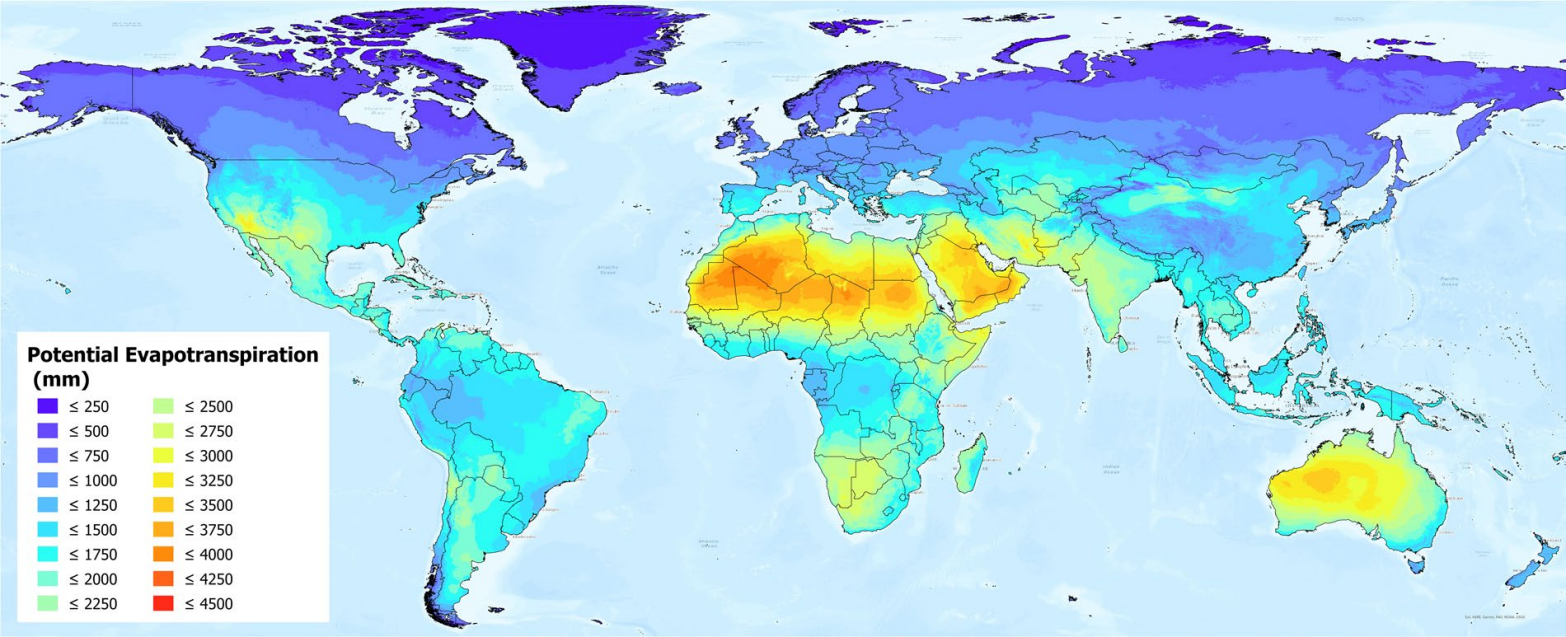
Global reference evapotranspiration (Global-ET_0__v3) calculated using the FAO-56 Penman Monteith equation for the entire globe at 1 km spatial resolution.

**Fig. 2:**
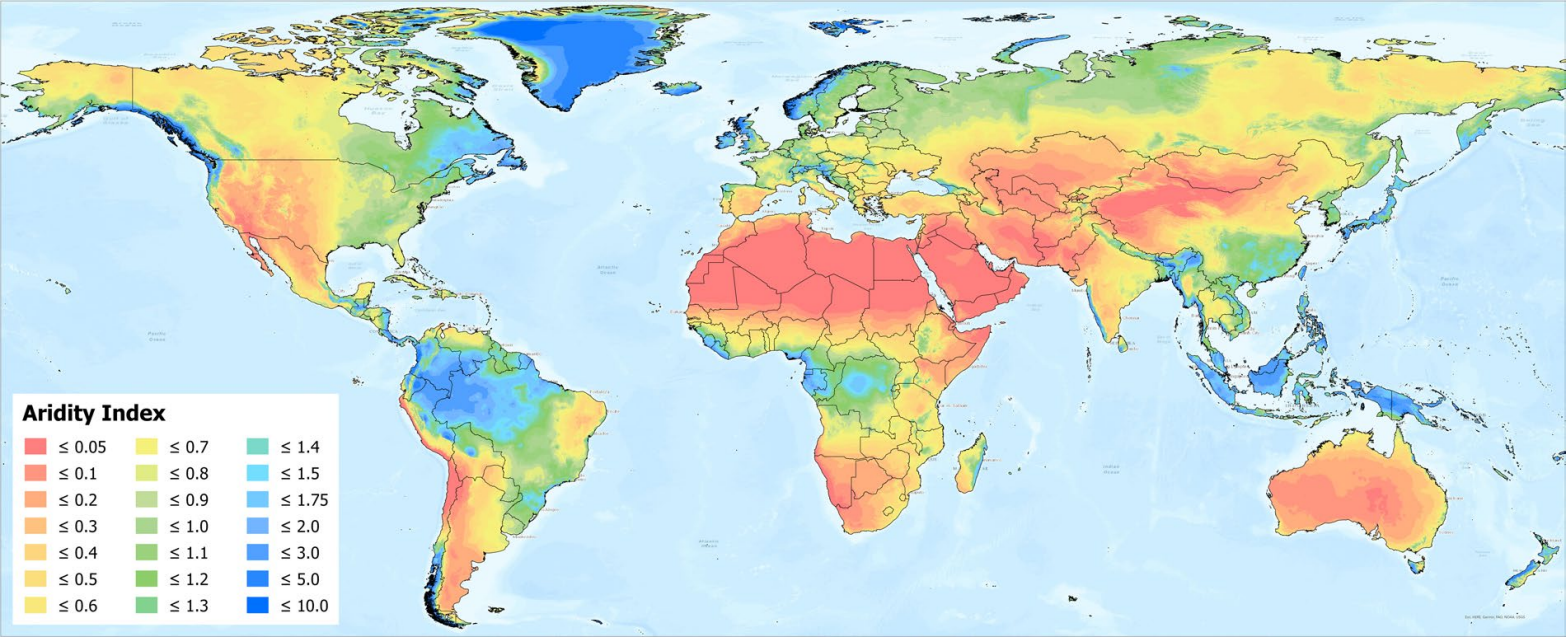
Global Aridity Index (Global-AI_v3), based upon the FAO-56 Penman Monteith equation for reference evapotranspiration (ET_0_) calculated for the entire globe. Note that higher AI_ET_0_ (green/blue colors) represents more humid conditions, with low AI (yellow/brown/red colors) representing higher aridity.

**Fig. 3:**
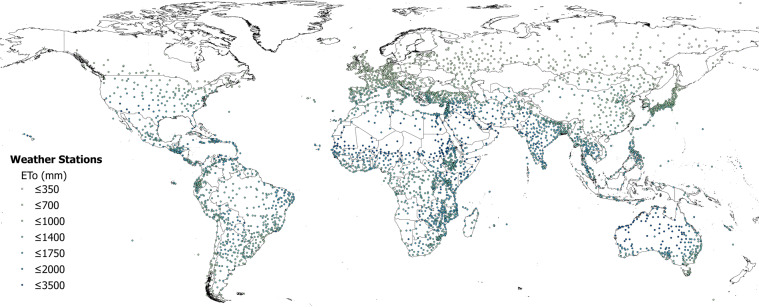
Location of weather stations included in the FAO CLIMWAT dataset, showing ET_0__CLIMWAT values for Penman-Monteith Reference Evapotranspiration (ET_0_).

The global estimations of ET_0_ and AI were first evaluated against the FAO “CLIMWAT 2.0 for CROPWAT”^47^ (Figs. 1 and 2) global database using long-term monthly mean values of climatic parameters derived from weather station data, roughly covering the period of 1970–2000, concurrent with the temporal coverage of the WorldClim version 2.0/2.1 database. CLIMWAT 2.0 provides observed agroclimatic data of over 5000 stations distributed worldwide (Fig. 3), including monthly averages for seven climatic parameters, namely maximum temperature, minimum temperature, relative humidity, wind speed, sunshine hours, radiation balance and ET_0_ calculated according to the Penman-Monteith method, as well as the coordinates and altitude of the station.

**Fig. 4:**
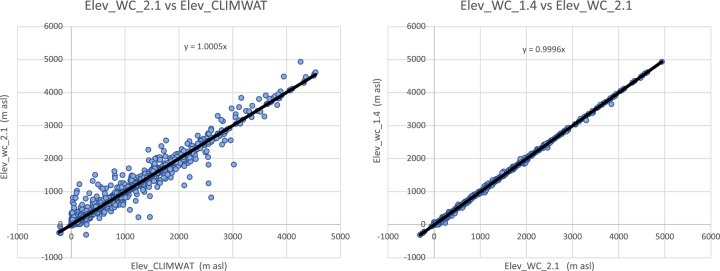
Validation and comparison of elevation data (m asl) used in the analysis, current and previous.

**Fig. 5:**
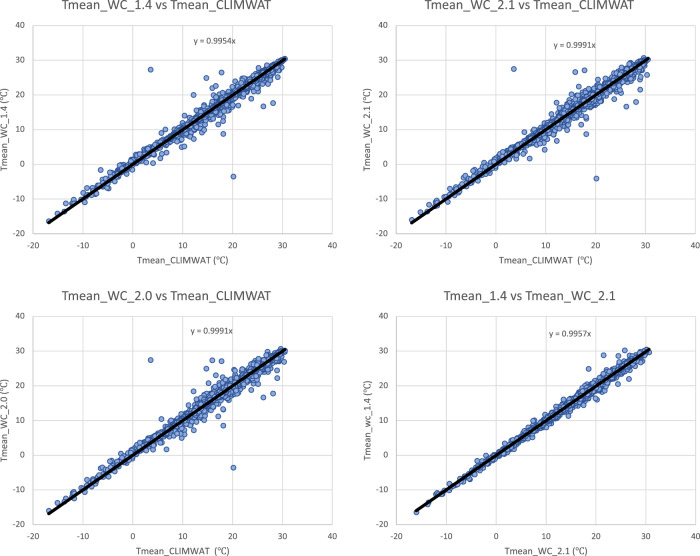
Validation and comparison of mean annual temperature data (°C) used in the analysis, current and previous.

**Fig. 6:**
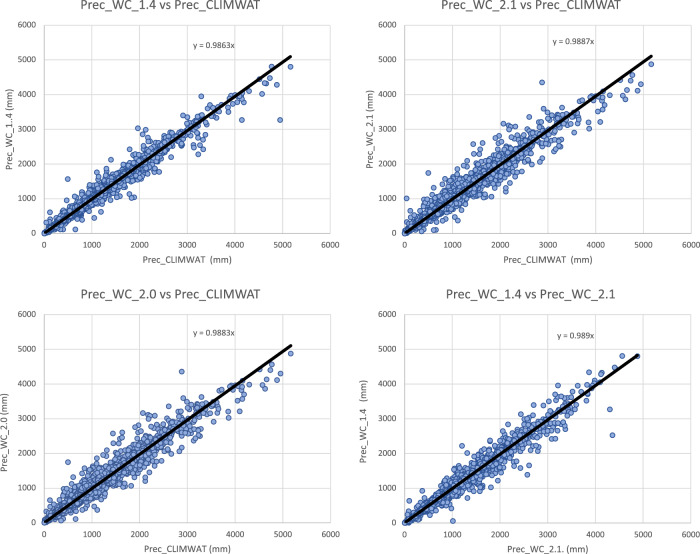
Validation and comparison of mean annual precipitation data (mm) used in the analysis, current and previous.

Input parameters from the three WorldClim spatial datasets (versions: 1.4; 2.0; 2.1) were compared with the values extracted from the weather station data to evaluate the accuracy and overlap of the CLIMWAT and WorldClim datasets, and the suitability of using the CLIMWAT to evaluate the performance of the ET_0_ spatial estimation, by sampling of the gridded data at the weather station coordinates. An assessment of the digital elevation data (DEM) provided by WorldClim 2.1, and used in our estimation, against that reported by CLIMWAT station data (Table 1; Fig. 4) showed a high level of accuracy (r^2^ = 0.98), providing some confidence in the locational accuracy of the weather station data. The elevation data we used in this current analysis was virtually identical (r^2^ = 1.00) to the DEM’s used in previous versions of the Global-AI_PET databases. Likewise, a comparison of mean annual temperature data revealed no significant differences in these datasets (r^2 ^ > 0.98 for all dataset comparisons), with the global average of each being nearly identical (≈ 17.8 °C) Fig. 5, indicating an absence of globally systematic bias towards over- or under-estimation of temperature. Annual precipitation as identified from the WorldClim 2.1 grids was also found to be highly correlated (r^2^ = 0.96) with that reported by the CLIMWAT weather station data (Table 1; Fig. 6), but with a moderately high stand error (148 mm), although more than WorldClim 1.4 (r^2^ = 0.98), which covered a different temporal span (1960–1990). A comparison of the average global mean annual precipitation (*MA_Prec*) between the CLIMWAT and the WorldClim v. 2.1 data showed identical results (990 mm), with version 1.4 averaging slightly less (984 mm). As the input parameters from the WorldClim 2.1 showed high levels of accuracy in comparison to the CLIMWAT data, we concluded that the CLIMWAT was an appropriate dataset available for evaluating the accuracy of the ET_0_ and AI estimation algorithms.

**Table 1:** Summary Table of Technical Validation Results.

Regression	R Square	Standard Error	Bias
**Elvevation**			
Elev_WC_2.1 ve Elev_Climwat	0.98	108	−3.73
Elev_WC_1.4 vs Elev_Climwat	0.98	108	−2.53
Elev_WC_1.4 vs ELev_WC_2.1	1.00	13	−1.22
**Mean Annual Temperature**			
Tmean_WC_1.4 vs Tmean_CLIMWAT	0.99	0.93	0.03
Tmean_WC_2.0 vs Tmean_CLIMWAT	0.98	1.02	−0.06
Tmean_WC_2.1 vs Tmean_CLIMWAT	0.98	1.01	−0.06
Tmean_WC_1.4 vs Tmean_WC_2.1	1.00	0.56	−0.10
**Mean Annual Precipitation**			
Prec_CLIMWAT vs Prec_WC_1.4	0.98	110	5.23
Prec_CLIMWAT vs Prec_WC_2.0	0.96	150	−0.95
Prec_CLIMWAT vs Prec_WC_2.1	0.96	148	−1.61
Prec_WC_1.4 vs Prec_WC_2.1	0.97	122	−6.84
**Potential Evapotranspiration_ET**_**0**_			
ET_0__CLIMWAT_XLS vs ET_0__CLIMWAT	0.99	36	−53.71
Global_PET_v1 vs ETo_CLIMWAT	0.72	221	−132.65
Global_ET_0__v2 vs ET_0__CLIMWAT	0.84	221	−385.75
**Global_ET**_**0**_**_v3 vs ET**_**0**_**_CLIMWAT**	**0.85**	**219**	**−389.38**
Global_ET_0__v1 vs Global_ET_0__v3	0.65	249	−256.73
ET_0__CLIMWAT vs ET_0__CRU_TS	0.84	160	−18.74
**Global_ET**_**0**_**_v3 vs ET**_**0**_**_CRU_TS**	**0.89**	**136**	**−408.12**
**Aridity Index**			
Global_AI_v1 vs AI_CLIMWAT	0.91	0.16	0.14
Global_AI_v2 vs AI_CLIMWAT	0.888	0.18	0.21
**Global_AI_v3 vs AI_CLIMWAT**	**0.90**	**0.17**	**0.21**
Global_AI_v1 vs Global_AI_v3	0.89	0.18	0.07
AI_CLIMWAT vs AI_CRU_TS	0.77	0.33	−0.02
**Global_AI_v3 vs AI_CRU_TS**	**0.83**	**0.22**	**0.23**
** Evaluated datasets	**Description**		
**Elevation**			
Elev_Climwat	Elevation data from CLIMWAT station data		
Elev_WC_1.4	Elevation data supplied with WC_1.4		
Elev_WC_2.1	Elevation data suppled with WC_2.0 and WC_2.1		
**Mean Annual Temperature**			
Tmean_ClimWat	Temperature data from CLIMWAT station data		
Tmean_WC_1.4	Temperature data from WC_1.4		
Tmean_WC_2.0	Temperature data from WC_2.0		
Tmean_WC_2.1	Temperature data from WC_2.1		
**Mean Annual Precipitation**			
Prec_ClimWat	Precipitation data from CLIMWAT station data		
Prec_WC_1.4	Precipitation data from WC_1.4		
Prec_WC_2.0	Precipitation data from WC_2.0		
Prec_WC_2.1	Precipitation data from WC_2.1		
**PET (ET**_**0**_**)**			
ET_0__ClimWat	ET_0_ as reported by CLIMWAT station data		
ET_0__ClimWat_XLS	ET_0_ calculated using estimation algorithm parameterized with CLIMWAT station data		
ET_0__CRU_TS	ET_0_ extracted from CRU_TS PET grid		
Global_PET_v1	PET calculated using WC_1.4 (Modified Hargreaves-Thornton)		
Global_ET_0__v2	ET0 calculated using WC_2.0 (Penman-Montieth)		
**Global_ET**_**0_**_**v3**	**ET0 calculated using WC_2.1 (Penman-Montieth)**		
**Aridity Index (AI)**			
AI_ClimWat	AI calculated using parameters from CLIMWAT station data (Penman-Montieth)		
AI_CRU_TS	AI calculated using CRU_TS (Penman-Montieth)		
Global_AI_v1	AI calculated using WC_1.4 (Modified Hargreaves-Thornton)		
Global_AI_v2	AI calculated using WC_2.0 (Penman-Montieth)		
** Global_AI_v3**	**AI calculated using WC_2.1 (Penman-Montieth)**

**Fig. 7:**
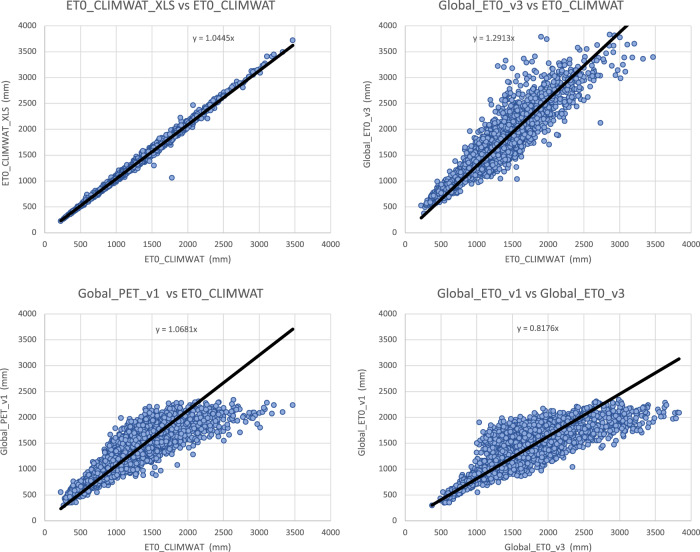
Validation and comparison of the ET_0_ estimates (mm) produced by the analysis, current and previous.

The calculation used to derive the ET_0_ estimation was tested against the ET_0_ estimates provided by CLIMWAT, using the CLIMWAT provided parameters from 4242 weather stations to parameterize the estimation algorithm (Table 1; Fig. 7). The calculated ET_0_ was shown to be highly accurate (r_2_ = 0.99) with a very low standard error (36 mm), providing confidence that the algorithm provides an accurate estimation. When the algorithm was implemented geospatially on a per grid cell basis to produce the Global_AI_PET_v3 dataset and tested against the CLIMWAT ET_0_ estimates from 3842 weather stations, the results showed a relatively high level of accuracy (r^2^ = 0.85), sufficient for use within many modeling and other scientific efforts. Local estimates, however, may have high variability associated with steep elevation gradients and heterogenous terrain, and/or low levels of accuracy at the grid cell level due to interpolation of scattered or less dense weather station data, as there is significant potential for error associated with the global input data.

**Fig. 8:**
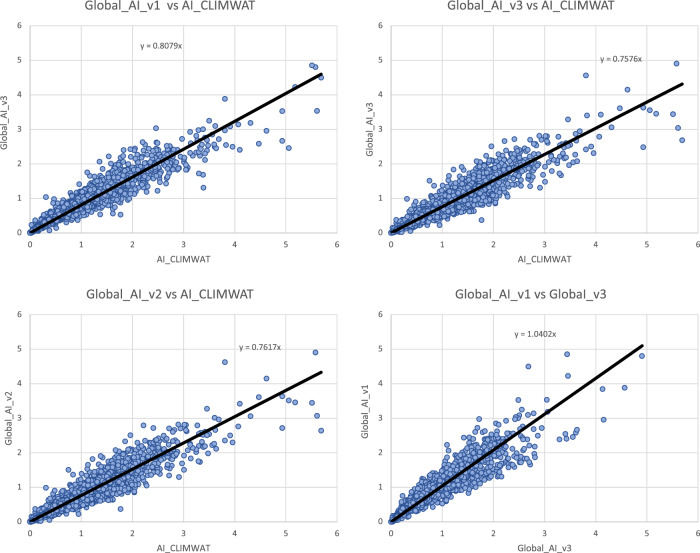
Validation and comparison of aridity index data produced by the analysis, current and previous. Values are unitless, with higher values indicating increasing moisture availability.

Whereas the ET_0_ based on the WorldClim 2.1 data was virtually identical to that produced by the WorldClim 2.0 (r^2^ = 1.00, std error = 27 mm), differences were more significant when compared with the previous Global-AI_PET_v1 of the PET estimation (r^2^ = 0.65). The ET_0_ estimates based on the latest version of the WorldClim (v. 2.1) showed a significant improvement over the Modified Hargreaves PET estimates of the Global-AI_PET_v2 (r^2^ = 0.85 vs r^2^ = 0.72), using WorldClim v. 1.4, with the Hargreaves methodology systematically underestimating higher PET values. Similarly, the AI estimates based on the Global-AI_PET_v3 analysis, when compared to AI estimates based on parameters provided by the CLIMWAT weather station data (Table 1; Fig. 8), showed a high level of correspondence (r^2^ = 0.90), statistically the same but nominally slightly less than from the Global-AI_PET_v1 estimates (r^2^ = 0.91).

**Fig. 9:**
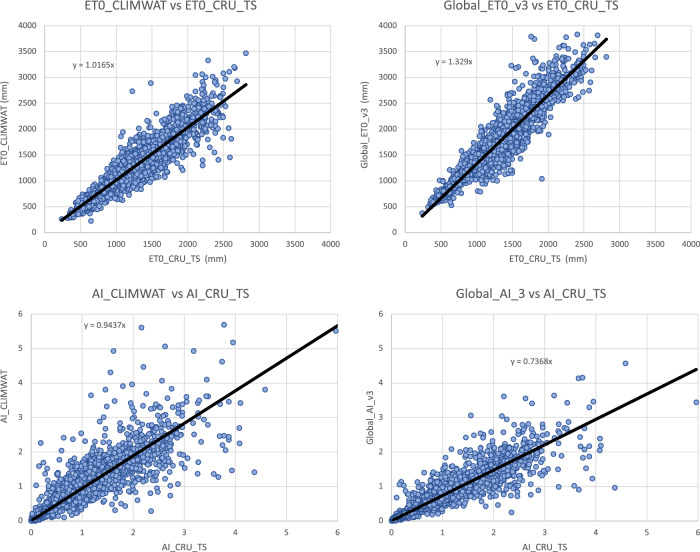
Validation and comparison of Et_0_ and AI results, with data and results from the CRU_TS (v. 4.04) dataset.

Similarly, the global estimations of ET_0_ were evaluated against the calculated PET (ET_0_) provided by the CRU_TS (Climatic Research Unit gridded Time Series version 4.05)^48^. The CRU_TS is a widely used climate dataset on a 0.5° latitude by 0.5° longitude grid over all land domains of the world except Antarctica. It is derived by the interpolation of monthly climate anomalies from extensive networks of weather station observations. PET values are provided in the CRU_TS dataset, calculated based upon the Penman-Monteith formula^25,26^, using the CRU_TS gridded values of mean temperature, vapour pressure, cloud cover and wind field. For our comparison, we averaged the CRU_TS monthly values for PET from 1971–2000 to obtain a global coverage of average annual PET for that time period. The same CLIMWAT meteorological stations used in the previous comparisons were used as sample points for the comparison with the latest version of the ET_0_ dataset (based on WorldClim v 2.1), and the CLIMWAT ET_0_ was also compared with the CRU_TS PET dataset (r^2^ = 0.84) to assess general congruence among the datasets (Fig. 9). The CRU_TS precipitation data for that time period was similarly averaged and used to calculate an AI based upon the CRU_TS dataset and compared to the Global-AI_PET_v3. Results showed a high level of agreement for both the ET_0_ and the AI comparison (r^2^ = 0.89; r^2^ = 0.83, respectively), considering the coarser resolution of the CRU_TS data is a likely source of error in the comparison with finer resolution data of the Global-AI_PET_v3.

Although we caution the users on the limitations of the data, we conclude with a high level of confidence that this revised ET_0_/AI dataset produced using our geospatially implemented algorithm based upon the FAO Penman-Monteith equation provides an adequate and usable global estimation of PET and AI suitable for a variety of non-mission critical applications, at scales from local, to national, regional, and global. Local topography, landscape heterogeneity, and interpolation of weather station networks all contribute to increasing error at more specific levels, such as plot or field level, especially in areas where weather station density is sparse. However, based upon this technical evaluation, the authors concur that this current version (Global-AI_PET_v3) dataset is improved over previous versions, with a high correlation to real world weather station data, and as such, find it to be a valuable publicly available global public good, with comparative advantage as a reference resource, and global coverage at 30 arc-second resolution. Developed using the agreed upon standard methodology for estimation of ET_0_, based upon FAO-56 Penman-Monteith, this dataset (and its source code) represents a robust tool for a variety of scientific investigations in an era of rapidly changing climatic conditions.

## Usage Notes

The geospatial datasets are provided online in GeoTIFF (.tif) format, in geographic coordinates; datum and spheroid are WGS84; spatial units are decimal degrees. The spatial resolution is 30 arc-seconds or 0.008333 degrees (approximately 1 km^2^ at the equator).

The Aridity Index (Global-AI) geodatasets have been multiplied by a factor of 10,000 to derive and distribute the data as integers (with 4 decimal accuracy). The AI values in the GeoTIFF (.tif) files need to be multiplied by 0.0001 to retrieve the values in the correct units.

## Data Availability

The Global ET0 and Aridity Index Database v3 (Global-AI_PET_v3)^46^ is archived on the Figshare Open Repository: 10.6084/m9.figshare.7504448.v5.

## Code availability

Geospatial processing and analysis were done using ESRI ArcGIS Pro (version 2.9), ArcMap (version 10.8), Python (versions 2.7 & 3.6) programming language, and Microsoft Excel for further data analysis, graphic and presentation. The Python programming code^66^ used to run the calculation of ET_0_ and AI is provided and available online at: 10.6084/m9.figshare.20005589.
